# Trends in the Incidence of New-Onset Anorexia Nervosa and Atypical Anorexia Nervosa Among Youth During the COVID-19 Pandemic in Canada

**DOI:** 10.1001/jamanetworkopen.2021.37395

**Published:** 2021-12-07

**Authors:** Holly Agostino, Brett Burstein, Dina Moubayed, Danielle Taddeo, Rosheen Grady, Ellie Vyver, Gina Dimitropoulos, Anna Dominic, Jennifer S. Coelho

**Affiliations:** 1 Division of Adolescent Medicine, Department of Pediatrics, Montreal Children’s Hospital, McGill University Health Centre, Montreal, Quebec, Canada; 2 Division of Pediatric Emergency Medicine, Department of Pediatrics, Montreal Children’s Hospital, McGill University Health Centre, Montreal, Quebec, Canada; 3 Department of Epidemiology, Biostatistics and Occupational Health, McGill University, Montreal, Quebec, Canada; 4 Section of Adolescent Medicine, Department of Pediatrics, Sainte Justine Hospital, Université de Montréal, Montreal, Quebec, Canada; 5 Division of Adolescent Medicine, Department of Pediatrics, McMaster’s Children’s Hospital, McMaster University, Hamilton, Ontario, Canada; 6 Section of Adolescent Medicine, Department of Pediatrics, Alberta Children's Hospital, University of Calgary, Calgary, Alberta, Canada; 7 Faculty of Social Work, Departments of Pediatrics and Psychiatry, University of Calgary, Calgary, Alberta, Canada; 8 Division of Adolescent Medicine, Department of Pediatrics, Janeway Children’s Hospital, Memorial University, St John’s, Newfoundland, Canada; 9 Department of Psychiatry, University of British Columbia, and Provincial Specialized Eating Disorders Program for Children and Adolescents, British Columbia Children’s Hospital, Vancouver, British Columbia, Canada

## Abstract

**Importance:**

The COVID-19 pandemic has had considerable mental health consequences for children and adolescents, including the exacerbation of previously diagnosed eating disorders. Whether the pandemic is a factor associated with the concomitant increase in new-onset anorexia nervosa or atypical anorexia nervosa remains unknown.

**Objective:**

To assess the incidence and severity of newly diagnosed anorexia nervosa or atypical anorexia nervosa in a national sample of youth before and during the first wave of the COVID-19 pandemic.

**Design, Setting, and Participants:**

This repeated cross-sectional study analyzed new eating disorder assessments that were conducted at 6 pediatric tertiary-care hospitals in Canada between January 1, 2015, and November 30, 2020. Patients aged 9 to 18 years with a new anorexia nervosa or atypical anorexia nervosa diagnosis at the index assessment were included.

**Exposures:**

COVID-19–associated public health confinement measures during the first wave of the pandemic (March 1 to November 30, 2020).

**Main Outcomes and Measures:**

Primary outcomes were the incidence and hospitalization rates within 7 days of de novo anorexia nervosa or atypical anorexia nervosa diagnosis. Event rate trends during the first wave were compared with trends in the 5-year prepandemic period (January 1, 2015, to February 28, 2020) using an interrupted time series with linear regression models. Demographic and clinical variables were compared using a χ^2^ test for categorical data and *t* tests for continuous data.

**Results:**

Overall, 1883 children and adolescents with newly diagnosed anorexia nervosa or atypical anorexia nervosa (median [IQR] age, 15.9 [13.8-16.9] years; 1713 female patients [91.0%]) were included. Prepandemic anorexia nervosa or atypical anorexia nervosa diagnoses were stable over time (mean [SD], 24.5 [1.6] cases per month; β coefficient, 0.043; *P* = .33). New diagnoses increased during the first wave of the pandemic to a mean (SD) of 40.6 (20.1) cases per month with a steep upward trend (β coefficient, 5.97; *P* < .001). Similarly, hospitalizations for newly diagnosed patients increased from a mean (SD) of 7.5 (2.8) to 20.0 (9.8) cases per month, with a significant increase in linear trend (β coefficient, −0.008 vs 3.23; *P* < .001). These trends were more pronounced in Canadian provinces with higher rates of COVID-19 infections. Markers of disease severity were worse among patients who were diagnosed during the first wave rather than before the pandemic, including more rapid progression (mean [SD], 7.0 [4.2] months vs 9.8 [7.4] months; *P* < .001), greater mean (SD) weight loss (19.2% [9.4%] vs 17.5% [9.6%]; *P* = .01), and more profound bradycardia (mean [SD] heart rate, 57 [15.8] beats per minute vs 63 [15.9] beats per minute; *P* < .001).

**Conclusions and Relevance:**

This cross-sectional study found a higher number of new diagnoses of and hospitalizations for anorexia nervosa or atypical anorexia nervosa in children and adolescents during the first wave of the COVID-19 pandemic in Canada. Research is needed to better understand the drivers and prognosis for these patients and to prepare for their mental health needs in the event of future pandemics or prolonged social isolation.

## Key Points

**Question:**

Is the COVID-19 pandemic associated with a change in the incidence and hospitalization rates for new-onset anorexia nervosa or atypical anorexia nervosa among youth?

**Findings:**

In this cross-sectional study of 1883 children and adolescents with newly diagnosed anorexia nervosa or atypical anorexia nervosa, the incidence of the disease increased from 24.5 to 40.6 cases per month and hospitalizations among these patients increased from 7.5 to 20.0 per month. During the first wave of the pandemic, the onset of illness was more rapid and disease severity was greater at presentation than before the pandemic.

**Meaning:**

Findings of this study suggest a need for expansion of eating disorder services as well as research to better understand the drivers and prognosis for this pediatric population.

## Teaser

This cross-sectional study compares the trends in incidence and hospitalization for eating disorders among children and adolescents 5 years before and in the first months of the COVID-19 pandemic.

## Introduction

The COVID-19 pandemic has had adverse implications for both the physical and mental health of children and adolescents worldwide.^1,2,3,4,5,6,7^ The World Health Organization declared COVID-19 a pandemic on March 11, 2020. Shortly after this declaration, public health mitigation strategies were mandated throughout Canada. By mid-March, Canadian provinces and territories had abruptly implemented, to varying degrees, school closures, prohibitions on gatherings, closures of nonessential businesses, and cancellation of sports and extracurricular activities. Public health authorities also cautioned against unnecessary visits to health care facilities to reduce viral transmission and to maintain capacity to accommodate surges in COVID-19 cases.^1^ Pediatric hospitals worldwide experienced decreased emergency department (ED) visits and hospital admissions throughout 2020.^2,3,4^ Despite the substantial reduction in the number of children and adolescents brought into hospitals for medical attention during the pandemic, numerous studies have reported increased pediatric mental health visits.^5,6,7^

The association between stressful events and exacerbations in eating disorder symptoms has been documented.^8^ Studies of adult patients with preexisting eating disorders reported worsening symptoms during the first wave of the COVID-19–associated confinement, including greater caloric restriction, increased self-induced vomiting, worsening body dysmorphia, and heightened exercise drive.^9,10,11^ Two single-center studies from Australia also found an increase in hospitalizations during the first wave of the pandemic among adolescents with previously diagnosed anorexia nervosa.^12,13^ Similarly, a recent single-center study in the US reported a doubling of hospitalizations for restrictive eating disorders during the COVID-19 pandemic.^14^ To date, the association between the pandemic and its confinement measures and the genesis of new-onset anorexia nervosa or atypical anorexia nervosa has not been studied. In this study, we sought to assess the incidence and severity of newly diagnosed anorexia nervosa or atypical anorexia nervosa in a national sample of children and adolescents before and during the first wave of the COVID-19 pandemic.

## Methods

This cross-sectional study was approved by the Research Ethics Board at each of the 6 participating institutions in Canada (Alberta Children’s Hospital, Calgary, Alberta; British Columbia Children’s Hospital, Vancouver, British Columbia; Janeway Children’s Hospital, St John’s, Newfoundland; McMaster Children’s Hospital, Hamilton, Ontario; Montreal Children’s Hospital, Montreal, Quebec; and Sainte Justine Hospital, Montreal, Quebec). Approval to waive direct patient consent was obtained from the Research Ethics Board at these sites for reasons of feasibility. We followed the Strengthening the Reporting of Observational Studies in Epidemiology (STROBE) reporting guideline.^15^

### Study Design, Setting, and Participants

We conducted a repeated cross-sectional analysis of all new eating disorder assessments between January 1, 2015, and November 30, 2020, at 6 of the 10 Canadian pediatric hospitals with tertiary-level eating disorder programs, which span 5 Canadian provinces from the western to the eastern coasts. Each of these 10 eating disorder programs were invited to participate in the study. At all of the participating study sites, the adolescent medicine service is involved with the assessment of consultations for youth younger than 18 years with symptoms that are suggestive of eating disorders. The referral sources for each program include community physicians, hospital subspecialists, and EDs that assess patients with acute presentations. Those who are diagnosed with an eating disorder and severely malnourished at the time of presentation are hospitalized for medical stabilization and nutritional rehabilitation. Admission criteria for patients with an eating disorder that are common to all study sites include full food refusal and/or evidence of substantial cardiovascular compromise (resting heart rate <50 beats per minute [bpm] and/or systolic blood pressure <90 mm Hg), as outlined by guidelines from the Society for Adolescent Health and Medicine.^16^ All 6 sites continued to conduct new eating disorder assessments throughout the study period (March 1 to November 30, 2020).

Patients were included in this study if they were aged 9 to 18 years and received a new diagnosis of anorexia nervosa or atypical anorexia nervosa, according to recognized definitions of the *Diagnostic and Statistical Manual of Mental Disorders* (Fifth Edition) (*DSM-5*). Because the *DSM-5* does not identify a specific weight threshold for atypical anorexia nervosa, patients with a percentage of median body mass index (BMI) that was greater than 85% at the time of assessment were classified as having atypical anorexia nervosa, as described elsewhere.^17^ Patients were excluded if they met the criteria for a *DSM-5* eating disorder other than anorexia nervosa or atypical anorexia nervosa (eg, avoidant restrictive food intake disorder, bulimia nervosa, and binge eating disorder) or if their diagnosis remained unclear after the initial assessment. Patients who were diagnosed with an eating disorder before the index assessment were also excluded from analysis. Metrics on race and ethnicity were not collected because these are not routinely included at Canadian databanks or medical records.

### Outcomes and Variables

The primary outcome measures were the incidence and hospitalization rates within 7 days of de novo anorexia nervosa or atypical anorexia nervosa diagnosis. Secondary outcomes included eating disorder–specific variables of interest that were obtained from patient medical records: self-reported duration of restrictive behaviors before assessment, vital signs at time of assessment, percentage of median BMI, percentage of body weight loss, and amount and rate of weight loss. Baseline demographic data were also collected, including age, sex assigned at birth, BMI at assessment, and index visit diagnosis. The World Health Organization growth curves for age and sex were used to calculate median BMI. Premorbid weight and amount and rate of weight loss were calculated according to the growth records at the time of the assessment. When not available, premorbid weight was based on self-reported information.

The main exposure was COVID-19–associated public health confinement measures. For the purpose of this analysis, the beginning of the pandemic period was defined as March 1, 2020, which corresponded with the earliest public health recommendations that slightly preceded the official provincial state-of-emergency declarations and lockdown measures across Canada (from March 13 to 27, 2020).^18^ The prepandemic period was defined as January 1, 2015, to February 28, 2020.

### Statistical Analysis

Incidence and hospitalization rates for all de novo anorexia nervosa or atypical anorexia nervosa diagnoses during the first wave of the pandemic were compared with rates in the 5-year prepandemic period. Monthly counts were plotted for the number of new cases and for the number of patients requiring medical hospitalization within 7 days of their index presentation or diagnosis.

We performed an interrupted time series analysis with linear regression modeling to estimate time trends (and 95% CIs) in national monthly event rates before and during the first wave of the pandemic as well as to identify the change in time trends associated with COVID-19 (March 2020) and the immediate implication of COVID-19 for the event rate (equivalent to the mean difference between pre- and post-COVID-19 event rates, controlling for time trends). In addition to pooled national-level analyses, site-specific charts were plotted and analyzed for each of the 6 study sites. Autocorrelation was assessed between months using Durbin-Watson tests and was further validated for the interrupted time series with the Newey-West adjustment for SE, which also demonstrated no correlation.

No data were missing for the primary or secondary analyses. Baseline demographic and clinical data for eating disorder–specific variables were presented in descriptive tables and were compared before and during the first wave of the pandemic using either a χ^2^ test for categorical data or an unpaired *t* test for continuous data. A 2-tailed *P* < .05 was considered statistically significant. All analyses were performed using SAS, version 9.4 (SAS Institute).

## Results

A total of 1883 children and adolescents (median [IQR] age, 15.9 [13.8-16.9] years; 1713 female [91.0%] and 170 male [9.0%] youth) who were newly diagnosed with anorexia nervosa or atypical anorexia nervosa across the 6 study sites were included in the analysis. The number of patients included from each site and their baseline characteristics before and during the first wave of the COVID-19 pandemic are shown in Table 1. Most patients were diagnosed with atypical anorexia nervosa both before (783 of 1538 [50.8%]) and during (175 of 345 [50.7%]) the first wave.

**Table 1.** Demographic Characteristics of Patients With Anorexia Nervosa or Atypical Anorexia Nervosa Diagnosed Before or During the COVID-19 Pandemic in Canada

	No. (%)	
	Diagnosis before COVID-19 pandemic (n = 1538)	Diagnosis during first wave of COVID-19 pandemic (n = 345)
Age, mean (SD), y^a^	14.9 (1.7)	14.9 (1.7)
Sex assigned at birth		
Female^a^	1399 (90.8)	314 (91.0)
Male^a^	139 (9.0)	31 (8.9)
Diagnosis		
Anorexia nervosa^a^	755 (49.1)	170 (49.2)
Atypical anorexia nervosa^a^	783 (50.8)	175 (50.7)
Participants by hospital site		
British Columbia Children’s Hospital	142 (9.2)	21 (6.1)
Alberta Children’s Hospital	291 (18.9)	33 (9.6)
McMaster Children’s Hospital	325 (21.1)	94 (27.2)
Sainte Justine Hospital	366 (23.8)	98 (28.4)
Montreal Children’s Hospital	281 (18.2)	72 (20.9)
Janeway Children’s Hospital	133 (8.6)	27 (7.8)

^a^ Baseline characteristics before vs during the pandemic had a *P* > .99.

The time trends of total newly diagnosed cases and hospitalizations per month in the pooled national sample are shown in the Figure. For the 5-year period preceding the pandemic, the time trend was stable over time (β coefficient, 0.043; *P* = .33), and the mean (SD) number of newly diagnosed cases during this period was 24.5 (1.6) cases per month (Table 2). During the first wave, newly diagnosed cases demonstrated a steep upward trend (β coefficient, 5.97; *P* < .001), and the mean (SD) cases during this period increased to 40.6 (20.1) cases per month (*P* < .001). Hospitalizations for new patients similarly increased sharply along with the pandemic (β coefficient, −0.008 vs 3.23; *P* < .001), with the mean (SD) cases increasing from 7.5 (2.8) cases per month to 20.0 (9.8) cases per month (*P* < .001).

**Figure.**
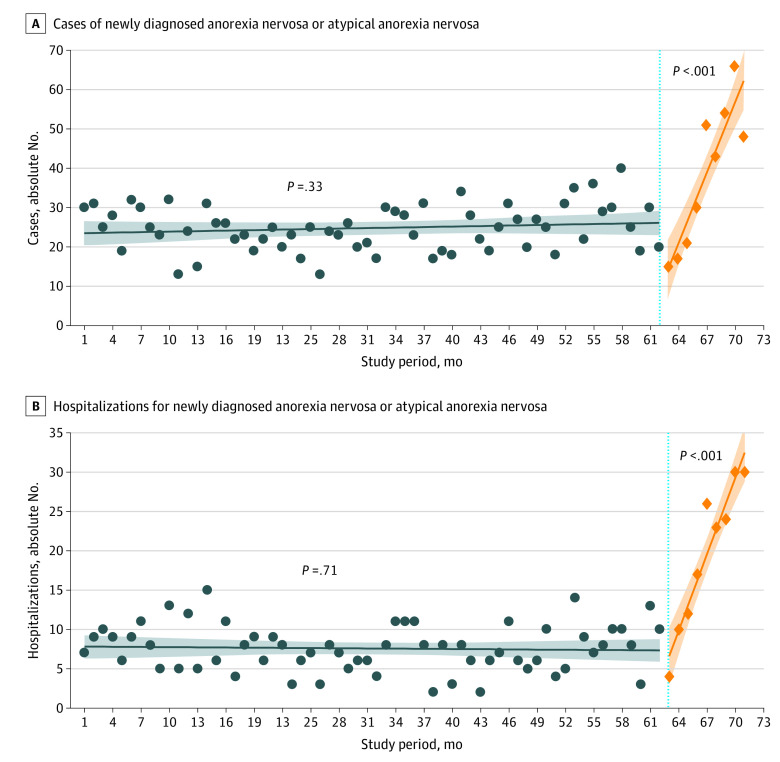
Interrupted Time Series of Newly Diagnosed Anorexia Nervosa or Atypical Anorexia Nervosa Cases and Hospitalizations per Month (with 95% CIs) in All Study Sites, January 1, 2015, to November 30, 2020 In Canada, the COVID-19 pandemic started in month 63 (March 1, 2020). The shading indicates the 95% CIs, blue circles, cases (A) and hospitalizations (B) per month before the first wave of the pandemic; blue line, prepandemic trend line of cases (A) and hospitalizations (B); orange diamonds, cases (A) and hospitalizations (B) per month during the first wave of the pandemic; orange line, trend line of cases (A) and hospitalizations (B) during the first wave of the pandemic.

**Table 2.** Cases and Hospitalizations for Anorexia Nervosa or Atypical Anorexia Nervosa Diagnosed Before or During the COVID-19 Pandemic in Canada

	No./mo, mean (SD)		*P* value
	Diagnosis before COVID-19 pandemic	Diagnosis during first wave of COVID-19 pandemic	
**Cases by site**			
National sample	24.5 (1.6)	40.6 (20.1)	<.001
British Columbia Children’s Hospital	2.3 (1.6)	2.6 (0.9)	.58
Alberta Children’s Hospital	4.7 (2.2)	4.1 (2.5)	.45
McMaster Children’s Hospital	5.1 (2.3)	10.4 (5.7)	<.001
Montreal Children’s Hospital	4.5 (2.3)	8.7 (5.9)	<.001
Sainte Justine Hospital	5.8 (2.2)	11.7 (6.7)	<.001
Janeway Children’s Hospital	2.1 (1.6)	3.1 (1.8)	.08
**Hospitalizations by site**			
National sample	7.5 (2.8)	20.0 (9.8)	<.001
British Columbia Children’s Hospital	1.4 (1.3)	1.9 (1.2)	.31
Alberta Children’s Hospital	0.9 (0.8)	2.3 (2.0)	.005
McMaster Children’s Hospital	2.2 (1.5)	6.6 (4.1)	<.001
Montreal Children’s Hospital	0.8 (1.0)	3.6 (2.6)	<.001
Sainte Justine Hospital	1.6 (1.3)	5.0 (3.6)	<.001
Janeway Children’s Hospital	0.6 (1.0)	0.78 (0.4)	.58

To assess for site-specific trends, new anorexia nervosa or atypical anorexia nervosa cases (eFigure 1 in the Supplement) and hospitalizations (eFigure 2 in the Supplement) were also analyzed by study site. Hospitals located in the Central Canadian provinces of Ontario and Quebec demonstrated the greatest increases in mean (SD) number of monthly cases (5.1 [2.3] to 10.4 [5.7] for McMaster Children’s Hospital; 4.5 [2.3] to 8.7 [5.9] for Montreal Children’s Hospital; and 5.8 [2.2] to 11.7 [6.7] for Sainte Justine Hospital) and monthly hospitalizations (2.2 [1.5] to 6.6 [4.1] for McMaster Children’s Hospital; 0.8 [1.0] to 3.6 [2.6] for Montreal Children’s Hospital; and 1.6 [1.3] to 5.0 [3.6] for Sainte Justine Hospital) during the first wave of the pandemic (Table 2). Upward linear trends at these sites most resembled the pooled national sample. Linear trends in hospitalizations at 2 additional sites (Alberta Children’s Hospital and Janeway Children’s Hospital) approached significance compared with prepandemic trends. Only 1 study site experienced no change in linear trend for new cases or hospitalizations compared with the prepandemic period (British Columbia Children’s Hospital).

To better understand the concomitant increase in hospitalizations, markers of anorexia nervosa severity were compared before and during the first wave of the pandemic for patients with newly diagnosed anorexia nervosa or atypical anorexia nervosa at all sites that reported an increase in newly diagnosed cases (Table 3). At these sites, all in Central Canada, patients with a new diagnosis during the pandemic had a shorter mean (SD) duration of restrictive symptoms (7.0 [4.2] months vs 9.8 [7.4] months; *P* < .001), with a higher mean (SD) percentage of body weight lost (19.2% [9.4%] vs 17.5% [9.6%]; *P* = .01) at a faster mean (SD) rate (2.1 [2.0] kg/mo vs 1.6 [1.7] kg/mo; *P* < .001). Moreover, these patients presented with more profound bradycardia at diagnosis (mean [SD] heart rate, 57 [15.8] bpm vs 63 [15.9] bpm; *P* < .001), with a greater proportion of patients meeting clinical criteria for admission compared with patients who were diagnosed before the pandemic (45.8% [121 of 264] vs 32.6% [317 of 972]; *P* < .001).

**Table 3.** Markers of Anorexia Nervosa or Atypical Anorexia Nervosa Severity at Presentation at Sites With an Increase in Newly Diagnosed Cases

	Mean (SD)		*P* value
	Diagnosis before COVID-19 pandemic (n = 972)	Diagnosis during first wave of COVID-19 pandemic (n = 264)	
Percentage of median BMI at diagnosis	89.4 (15.1)	88.6 (15.3)	.39
Weight loss, kg	10.5 (8.6)	11.7 (9.2)	.047
Length of illness, mo	9.8 (7.4)	7.0 (4.2)	<.001
Percentage of body weight lost	17.5 (9.6)	19.2 (9.4)	.01
Rate of weight loss, kg/mo	1.6 (1.7)	2.1 (2.0)	<.001
Heart rate at assessment, bpm	63 (15.9)	57 (15.8)	<.001
Systolic blood pressure at assessment, mm Hg	101.0 (12.2)	98.2 (11.9)	<.004
Patients with vital signs meeting criteria for hospitalization at assessment, No. (%)	317 (32.6)	121 (45.8)	<.001
Patients hospitalized at or within 1 wk of assessment, No. (%)	279 (28.7)	131 (49.6)	<.001

Abbreviations: BMI, body mass index; bpm, beats per minute.

## Discussion

To our knowledge, this study was the first to evaluate the association between the COVID-19 pandemic and new-onset anorexia nervosa or atypical anorexia nervosa in any patient population. In a broad national sample of youth who underwent an assessment in a tertiary care setting during the first wave of the pandemic, monthly cases of new-onset anorexia nervosa or atypical anorexia nervosa increased by more than 60% (24.5 to 40.6), and monthly hospitalizations nearly tripled (7.5 to 20.0) compared with prepandemic rates. Linear trends for both new cases and hospitalizations increased sharply, concomitant with pandemic confinement measures that began in March 2020. At sites with the greatest number of new cases, patients who received a diagnosis during the pandemic presented with more rapidly evolving and more severe markers of disease, which likely explains the observed increase in hospitalizations.

The largest increase in both new diagnoses and hospitalizations of anorexia nervosa or atypical anorexia nervosa was reported in Central Canada provinces (Quebec and Ontario). In these provinces, cases of COVID-19 and related mortality per capita were the highest during the first wave of the pandemic,^18^ and therefore the most restrictive confinement measures were adopted.^19^ In contrast, Western Canada reported relatively small growth in COVID-19 cases, and few cases were reported in Atlantic Canada.^19^ These findings suggested that provinces with high cases of COVID-19 infections and stricter confinement measures experienced a higher burden of newly diagnosed anorexia nervosa and atypical anorexia nervosa.

Provincial variations in the management of health services (eg, closures of primary care practices) may also be a factor in the differences between sites in the number of eating disorder assessments that were performed during the first wave of the pandemic. Some studies reported increases in acute mental health presentations at the ED^6,7^; however, it is unclear how these observations can be interpreted in the context of the pandemic. During the first wave, pediatric hospitals experienced dramatic decreases in ED visits and hospitalizations.^3,4^ Mental health presentations increased as a proportion of these visits,^20^ but not necessarily in absolute number. Moreover, in many provinces in Canada, public health authorities also mandated the closure of primary care clinics, limiting access to other mental health community resources. Therefore, analyses of ED visits and hospitalizations from the ED for anorexia nervosa or atypical anorexia nervosa may overestimate the true incidence. A strength of the present study was the inclusion of eating disorder assessments from a broad catchment of referral sources (outpatient clinics, inpatient wards, and EDs) at all study sites. As such, this study likely more accurately estimated the true burden of new-onset anorexia nervosa or atypical anorexia nervosa during the first wave than analyses of ED visits and hospitalizations alone.

The increase in new anorexia nervosa or atypical anorexia nervosa diagnoses during the first wave of the COVID-19 pandemic is likely multifactorial. Patients who were previously diagnosed reported experiencing worsening symptoms because of the pandemic.^9,10,11^ A recent single-center study at an adolescent eating disorder program found that, across all eating disorders, 40% of newly diagnosed patients cited the pandemic as a trigger for their eating disorder.^21^ Interviews conducted with adults with an eating disorder revealed an exacerbation of symptoms that was associated with increased anxiety, social isolation, and reduced contact with their treatment teams.^9,22^ Moreover, adults also reported worsening eating disorder symptoms in conjunction with a lack of distractions and constant exposure to stressful messages on social media.^23^ It is possible that these same stressors play a role in new-onset anorexia nervosa or atypical anorexia nervosa among children and adolescents.

COVID-19–associated restrictions varied in intensity and duration across Canada, but all provinces initially closed schools and nonessential businesses and canceled extracurricular activities. These changes had substantial consequences for eating, physical activity, and social patterns of adolescents, each of which may be a risk factor for developing anorexia nervosa cognitions.^23^ Lack of a clear routine may be associated with a higher risk for eating disorder–related behaviors because it removes structures that normalize eating. Confinement orders limit access to regular physical activity, which, in combination with disrupted eating patterns, may have a role in the heightened concern about body shape and weight.^24^ In addition, school closures likely expand social media use as a means of communication with peers. Media use has been associated with an increased risk for disordered eating, in particular through exposure to thin ideals and diet-related content.^25^ Furthermore, social media trends referring to weight gain during confinement and a focus on home cooking and exercise routines may have further elevated the eating disorder risk among youth.

Many adolescents with an eating disorder also have comorbid psychopathology, including depression, anxiety, and obsessive-compulsive disorder.^26^ Evidence suggests that the COVID-19 pandemic has had detrimental consequences for the mental health of both youths and their parents.^5,6,7,27^ Rates of depression and suicidal ideation were higher in adults in COVID-19–associated lockdowns compared with those who were not under these restrictions.^28^ In children and adolescents, the disruption of routines and disconnection from peers were associated with the increase in mental health burden and emergence of depression and anxiety.^20,29^ A worsening of overall mental health status may explain the increased rate of newly diagnosed anorexia nervosa or atypical anorexia nervosa found in the present study.

Protective factors against eating disorders in youth were also disrupted by the COVID-19 pandemic. Social support has been identified as a protective factor during stressful periods and as key to managing and reducing disordered eating.^30^ During the first wave of the pandemic, most countries used social distancing measures as a primary public health mitigation strategy. Many children and adolescents, who rely on peer group validation and connectedness, lost a primary source of social support that made them more vulnerable to stressful circumstances. In addition, access to primary care and routine screening was reduced or limited to virtual care. A consensus panel recently issued its recommendations for in-person medical evaluation for eating disorders when needed to ensure the appropriate assessment of medical instability.^31^ Reduced in-person medical assessments would likely make early detection of disordered eating more challenging and impede the early deployment of therapeutic interventions to slow or stop the progression of illness.

### Limitations

This study has some limitations. We were unable to make concrete causal inferences because factors other than the COVID-19 pandemic may have been associated with the increase of new-onset anorexia nervosa or atypical anorexia nervosa during the study period. Data collection occurred in 6 of the 10 pediatric tertiary eating disorder programs in Canada, spanning both coasts and including sites from the 4 most populous provinces (representing nearly 90% of the Canadian populace). However, not every province was represented, and all study sites are located in urban centers; thus, the findings may not be generalizable to all practice settings. All 6 study sites continued to see new eating disorder assessments during the first wave of the pandemic, and no site limited the number of hospitalizations. However, because of growing patient volumes, all sites shifted to prioritize the evaluation of youth with more severe presentations (eg, large amount of weight loss, substantial change in vital signs, or need for hospitalization); therefore, the possibility of selection bias cannot be excluded. Conversely, fewer youth with anorexia nervosa or atypical anorexia nervosa may have been identified and referred for assessment because of school closures, and many children and adolescents and their families may have chosen to avoid hospital centers for fear of exposure to COVID-19 infection. These factors suggest that the findings may be an underestimation of the true burden of newly diagnosed anorexia nervosa or atypical anorexia nervosa. This study only included patients who met the *DSM-5* criteria for anorexia nervosa or atypical anorexia nervosa at the initial assessment and therefore does not capture the total incidence and hospitalization rates across all eating disorder diagnoses. Other centers may have reached clinical significance if all eating disorder diagnoses were included. The degree of social confinement and school closures varied temporally between provinces, with Central Canada adopting longer and stricter confinement measures during the study period. The second wave of the pandemic (September 1 to December 31, 2020), which affected provinces more uniformly, was unlikely to be captured in these results and may have had a greater impact in provinces outside of Central Canada.

## Conclusions

In this cross-sectional study, we found an increase in the incidence and hospitalization rates of newly diagnosed anorexia nervosa or atypical anorexia nervosa in a national sample of youth during the first wave of the COVID-19 pandemic in Canada. Patients who were diagnosed in this period were more likely to present with rapidly evolving and more severe markers of disease. These findings highlight the need for expanded eating disorder and mental health programs during and after the COVID-19 pandemic. Research is still needed to better understand the drivers and prognosis for these patients and how best to prepare for their mental health needs in the event of future pandemics or prolonged social isolation.

## Notes

Supplement. **eFigure 1.** Interrupted Time Series of New Anorexia Nervosa/Atypical Anorexia Nervosa Cases per Month (With 95% Confidence Interval) by Study Site, January 2015 to November 2020 **eFigure 2.** Interrupted Time Series of Hospitalizations for New Anorexia Nervosa/Atypical Anorexia Nervosa per Month (With 95% Confidence Interval) by Study Site, January 2015 to November 2020
