# Supplementary material for: Trends in the Incidence of New-Onset Anorexia Nervosa and Atypical Anorexia Nervosa Among Youth During the COVID-19 Pandemic in Canada
Source: JAMA Netw Open. 2021 Dec 7;4(12):e2137395. doi: 10.1001/jamanetworkopen.2021.37395 (PMC8652595; doi:10.1001/jamanetworkopen.2021.37395)
Supplement: Supplement. — eFigure 1. Interrupted Time Series of New Anorexia Nervosa/Atypical Anorexia Nervosa Cases per Month (With 95% Confidence Interval) by Study Site, January 2015 to November 2020 eFigure 2. Interrupted Time Series of Hospitalizations for New Anorexia Nervosa/Atypical Anorexia Nervosa per Month (With 95% Confidence Interval) by Study Site, January 2015 to November 2020 [file jamanetwopen-e2137395-s001.pdf]

Supplementary Online Content

Agostino H, Burstein B, Moubayed D, et al. Trends in the incidence of new-onset anorexia nervosa and atypical anorexia nervosa among youth during the COVID-19 pandemic in Canada. *JAMA Netw Open*. 2021;4(12):e2137395. doi:10.1001/jamanetworkopen.2021.37395

eFigure 1. Interrupted Time Series of New Anorexia Nervosa/Atypical Anorexia Nervosa Cases per Month (With 95% Confidence Interval) by Study Site, January 2015 to November 2020

eFigure 2. Interrupted Time Series of Hospitalizations for New Anorexia Nervosa/Atypical Anorexia Nervosa per Month (With 95% Confidence Interval) by Study Site, January 2015 to November 2020

This supplementary material has been provided by the authors to give readers additional information about their work.

© 2021 Agostino H et al. *JAMA Network Open.*

eFigure 1. Interrupted Time Series of New Anorexia Nervosa/Atypical Anorexia Nervosa Cases per Month (With 95% Confidence Interval) by Study Site, January 2015 to November 2020

BC Children's Hospital

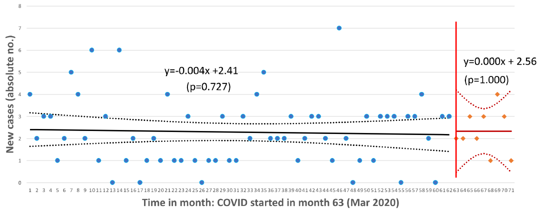

Alberta Children's Hospital

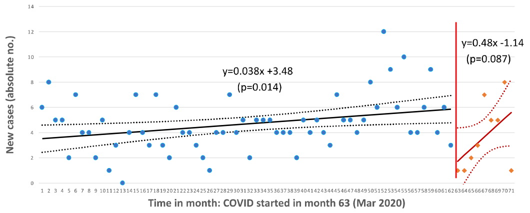

McMaster Children's Hospital

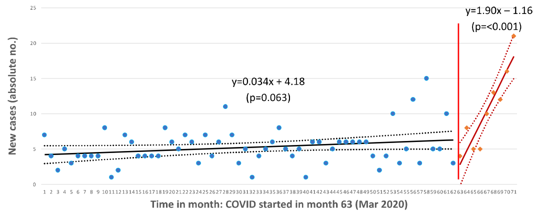

Sainte Justine Hospital

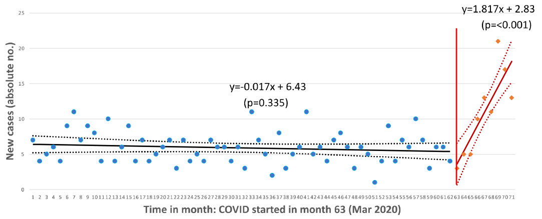

Montreal Children's Hospital

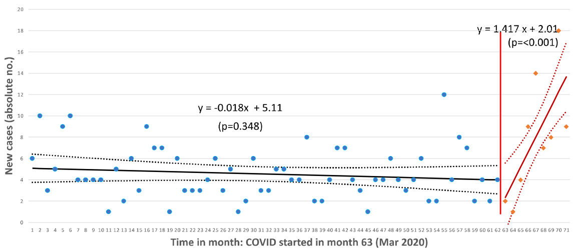

Janeway Children's Hospital

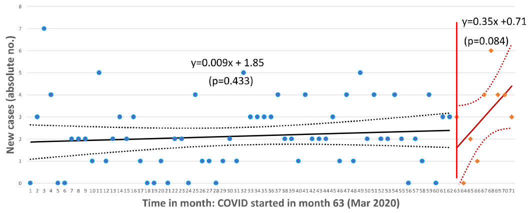

© 2021 Agostino H et al. *JAMA Network Open.*

eFigure 2. Interrupted Time Series of Hospitalizations for New Anorexia Nervosa/Atypical Anorexia Nervosa per Month (With 95% Confidence Interval) by Study Site, January 2015 to November 2020

BC Children's Hospital

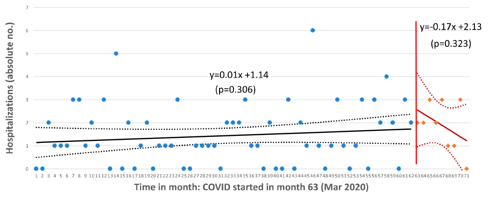

Alberta Children's Hospital

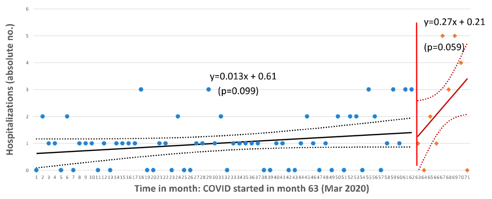

McMaster Children's Hospital

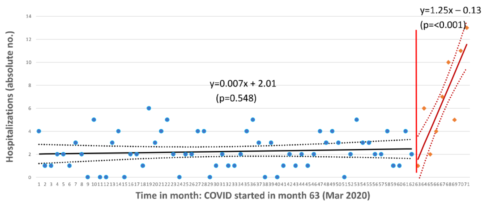

Sainte Justine Hospital

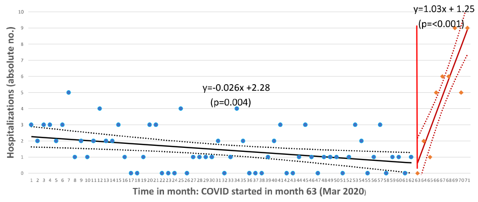

Montreal Children's Hospital

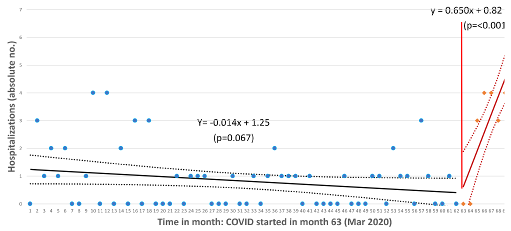

Janeway Children's Hospital

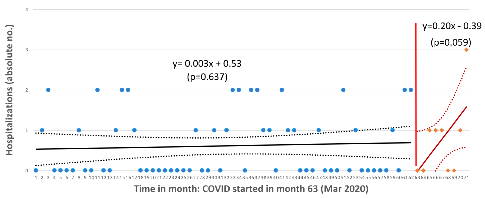

© 2021 Agostino H et al. *JAMA Network Open.*
